# Assessment of differentially private synthetic data for utility and fairness in end-to-end machine learning pipelines for tabular data

**DOI:** 10.1371/journal.pone.0297271

**Published:** 2024-02-05

**Authors:** Mayana Pereira, Meghana Kshirsagar, Sumit Mukherjee, Rahul Dodhia, Juan Lavista Ferres, Rafael de Sousa

**Affiliations:** 1 AI for Good Research Lab, Microsoft, Redmond, Washington, United States of America; 2 Department of Electrical Engineering, University of Brasilia, Brasilia, Brazil; 3 INSITRO, San Francisco, CA, United States of America; TTU: Tan Tao University, VIET NAM

## Abstract

Differentially private (DP) synthetic datasets are a solution for sharing data while preserving the privacy of individual data providers. Understanding the effects of utilizing DP synthetic data in end-to-end machine learning pipelines impacts areas such as health care and humanitarian action, where data is scarce and regulated by restrictive privacy laws. In this work, we investigate the extent to which synthetic data can replace real, tabular data in machine learning pipelines and identify the most effective synthetic data generation techniques for training and evaluating machine learning models. We systematically investigate the impacts of differentially private synthetic data on downstream classification tasks from the point of view of utility as well as fairness. Our analysis is comprehensive and includes representatives of the two main types of synthetic data generation algorithms: marginal-based and GAN-based. To the best of our knowledge, our work is the first that: (i) proposes a training and evaluation framework that does not assume that real data is available for testing the utility and fairness of machine learning models trained on synthetic data; (ii) presents the most extensive analysis of synthetic dataset generation algorithms in terms of utility and fairness when used for training machine learning models; and (iii) encompasses several different definitions of fairness. Our findings demonstrate that marginal-based synthetic data generators surpass GAN-based ones regarding model training utility for tabular data. Indeed, we show that models trained using data generated by marginal-based algorithms can exhibit similar utility to models trained using real data. Our analysis also reveals that the marginal-based synthetic data generated using AIM and MWEM PGM algorithms can train models that simultaneously achieve utility and fairness characteristics close to those obtained by models trained with real data.

## 1. Introduction

Differential privacy (DP) is the standard for privacy-preserving statistical summaries [1]. Companies such as Microsoft [2], Google [3], Apple [4], and government organizations such as the US Census [5], have successfully applied DP in machine learning [6, 7] and data sharing scenarios. The popularity of DP is due to its strong mathematical guarantees. Differential Privacy guarantees privacy by ensuring that the inclusion or exclusion of any particular individual does not significantly change the output distribution of an algorithm.

In areas ranging from health care, humanitarian action, education, and socioeconomic studies, the publication and sharing of data is crucial for informing society and scientific collaboration. However, the disclosure of such datasets can often reveal private, sensitive information. Privacy-preserving data publishing aims at enabling such collaborations while preserving the privacy of individual entries in the dataset. Tabular/categorical data about individuals are relevant in many applications, from health care to humanitarian action. Privacy-preserving data publishing for such data can be done in the form of a synthetic data table that has the same schema and similar distributional properties as the real data. The aim here is to release a perturbed version of the original information, so that it can still be used for statistical analysis, but the privacy of individuals in the database is preserved.

The biggest advantage of synthetic datasets is that, once released, all data analysis and machine learning tasks are performed in the same way it is done with real data. As noted by [8], the switch between real and synthetic data in data analysis and machine learning pipelines is seamless—the same analysis tools, libraries and algorithms are applied in the same manner in both datasets. Other privacy-preserving technologies, such as federated learning, requires expertise and appropriate tools to perform data analysis and model training.

Due to all the potential benefits of synthetic data, understanding the impacts of synthetic data in downstream classification tasks have become of extreme importance. A trend observed in recent studies is to evaluate performance of synthetic data generators of two types: marginal-based synthesizers [9] and generative adversarial networks (GAN) based synthesizers [8, 10, 11]. Marginal-based synthetic data generators are suitable for tabular data only, and have gained increased popularity after the algorithm MST won the NIST competition in 2018 [12]. Marginal-based synthesizers are named as such due to the fact that they learn approximate data distributions by querying noisy marginals from the real data. Notable marginal-based algorithms are MST [12], MWEM PGM [13], AIM [14] and PrivBayes [15]. GAN-based synthesizers, on the other hand, are flexible algorithms, and are suitable for tabular, image and other data formats. GANs learn patterns and relationships from the input data based on a game, in the sense of game theory, between two machine learning models, a discriminator model and the generator model. Among popular differentially private GAN architectures we list DP-GAN [16], DP-CTGAN [17], PATE-GAN [18] and PATE-CTGAN [17].

One of the major applications of synthetic data is for training machine learning models. Therefore, it is paramount to understand how exchanging real data for synthetic data impacts the performance of the trained machine learning models. By performance, we mean not only the utility of the model (its accuracy, for example) but also how well the model performs for different subgroups of the dataset—the fairness of the model. The impact of machine learning models on minorities subgroups is an active area of research, and several works have investigated the trade-offs among model accuracy, bias, and privacy [19–22]. However, only recently bias caused by the use of synthetic data in downstream classification received attention [9, 23, 24]. This problem becomes particularly relevant in the context of synthetic datasets generated with differential privacy guarantees. It is known that differential privacy can affect fairness in machine learning models [20]. Despite recent work investigating the impact of synthetic data in downstream model fairness [10, 23], there are important questions that remain unanswered:

- There is no published work that systematically studies the utility and fairness of machine learning models trained on several GAN based and marginal-based synthetic tabular dataset generation algorithms;
- Previous studies have not evaluated machine learning models trained on synthetic dataset generation algorithms for multiple definitions of fairness;
- In previous studies, it was always assumed that real data was available for evaluating the fairness of models trained on synthetic data. Here, we propose and evaluate a pipeline where no such assumption is necessary.

### 1.1 Contributions

In this work, we investigate the impacts of differentially private synthetic data on downstream classification, where we focus on understanding the impacts on model utility and fairness. Our investigation focus on two aspects of such impact:

- What is the impact in model utility when utilizing synthetic data for training machine learning models? Can synthetic data also be used to evaluate utility of machine learning models?
- What is the impact in model fairness when utilizing synthetic data for training machine learning models? Can synthetic data be used to evaluate fairness of machine learning models?

In our investigations we also evaluate if there are clear differences in performance between marginal-based and GAN-based synthetic data, and if there is a synthesizer algorithm type that produces data that clearly outperform others.

Our research work evaluates the impact of utilizing synthetic datasets for both training and testing in machine learning pipelines. We empirically compare the performance of marginal-based synthesizers and GAN-based synthesizers within the context of a machine learning pipeline for classification tasks. Our experiments yield a comprehensive analysis, encompassing utility and fairness metrics. Our main contributions are:

- We propose a training and evaluation framework that does not assume that real data is available for testing the utility and fairness of machine learning models trained on synthetic data.
- We present an extensive analysis of synthetic dataset generation algorithms in terms of privacy-loss, utility and fairness when used for training machine learning models. In particular, this is the first systematic comparison of several marginal-based and GAN-based algorithms for fairness and utility of the resulting machine learning models.
- This is the first of such studies that includes several different definitions of fairness.

### 1.2 Main findings

1. **Marginal-based synthetic data can accurately train machine learning models for tabular data.** Marginal-based synthetic data can train models with similar utility to models trained on real data. Our experiments show that for a privacy-loss parameter *ϵ* > 5.0, models trained with AIM (AUC = 0.683), MWEM PGM (AUC = 0.684), MST (AUC = 0.662) and Privbayes (AUC = 0.668) provides utility very similar to models trained on real data (AUC = 0.684). Additionally, we evaluated models using synthetic data, and found that marginal-based synthetic provides a good evaluation, with synthetic data providing an AUC = 0.666 versus AUC = 0.684 (measured using real data).
2. **Synthetic datasets generated with AIM and MWEM PGM have the potential be used for accurate model training and fairness evaluation in the case of tabular data.** Our experiments show that AIM and MWEM PGM synthetic data can train models that achieves very similar utility and fairness characteristics of models trained with real data. Additionally, the synthetic data generated by AIM algorithm, in our experiments, showed very similar behavior to real data when used to evaluate utility and fairness of machine learning models. This is the first study that presents evidence, from the perspective of utility and fairness, that synthetic data can be a substitute for real datasets in end-to-end machine learning pipelines for tabular data. It is interesting to investigate how these results generalize to larger data sets.

This work significantly extends and sub sums a previous version, presented at the *Machine Learning for Data: Automated Creation, Privacy, Bias Workshop* at the *International Conference on Machine Learning (ICML)* (workshop without proceedings) [25].

## 2. Related work

Synthetic data generation is a promising practice for privacy-preserving data sharing and publishing, understanding the impacts of utilizing synthetic data in machine learning pipelines is of significant importance. Although previous works have advised against using synthetic data to train and evaluate any final tools deployed in the real world [26], in very sensitive scenarios, such as human trafficking data [27], and electronic health records [28, 29], synthetic data is seen as a way to drastically increase the availability of research data. Particularly in health care, synthetic data can unlock research in areas like etiology of diseases, personalization of medicine, and healthcare administration assessment.

The promises synthetic data brings generated an interest in understanding impacts of utilizing synthetic in data analysis and machine learning. Some of these works include analyzing the utility of differentially private synthetic data in different tasks [30], investigating if training models with differentially private synthetic images can increase subgroup disparities [10], the impacts different types of synthetic data can have in model fairness [23], utility of synthetic data in downstream health care classification systems [9], and whether feature importance can be accurately analyzed using differentially private synthetic data [24]. The evaluation of impacts of synthetic datasets in machine learning pipelines is made by comparing models trained with real data with models trained on synthetic data. The comparison is performed by testing both models on real data. The comparison can be performed using utility metrics (AUC-ROC, F1-score, accuracy) and also fairness metrics (subgroup accuracy, statistical parity, equality of odds). A complete survey of evaluation metrics for synthetic datasets can be found in [28, 29].

Many of these works have made important findings in impacts of synthetic data in model utility and algorithmic fairness. In [30] a comparison among different types of differentially private synthetic data generation algorithms found that marginal-based algorithms outperform all other types of DP synthetic data generators when training machine learning classifiers, with performance nearly matching the performance of a classifier trained on real data. The paper [23] finds that marginal-based synthetic data (PrivBayes) impacts machine learning pipelines by decreasing model bias, while GAN-based synthetic data increases model bias. All these works are ultimately trying to answer the same question: to which extent can we substitute real data with synthetic data, and which are the best synthetic data generation techniques for model training?

However these works still left questions unanswered. First of all, there hasn’t been a systematic study of impacts of using synthetic datasets in end-to-end machine learning pipelines, which means evaluating the use of synthetic data for model training and model evaluation. Additionally, there has been a lot of focus on image classification tasks [10, 23] where the disparity in accuracy are largely attributable to the class imbalance in these datasets: i.e disadvantaged classes are also rare classes in the dataset thereby leading to worse performance on these. In contrast, our work studies these issues in the context of tabular datasets and in settings where the data has an intrinsic bias against sub-populations that are not necessarily rare in the dataset. We summarize in Table 1 how previous works have evaluated the impacts of synthetic tabular data in machine learning pipelines, and how our work differentiates from previous analysis. Although several works have assessed the performance of machine learning models trained with synthetic datasets [23, 30], this is the first study to analyze if synthetic datasets can be used for model assessment, and how close to reality such assessment is from the point of view of utility and fairness. Moreover, our work focus on comparing two types of data synthesizing algorithm families: marginal-based and GAN-based data synthesizers. While, these two type of data synthesizing algorithms have been previously compared for utility [30], no such extensive comparative analysis exists for fairness.

**Table 1:** Previous works evaluating differentially private synthetic data generation in machine learning pipelines for tabular data. The works presented in this table all focus on understanding the impact of utilizing differentially private synthetic datasets in machine learning pipelines either from a perspective of utility or from a perspective of algorithmic fairness.

PUBLICATION	EVALUATION OF SYNTHETIC DATA		EVALUATION OF ALGORITHMIC FAIRNESS
	AS TRAINING DATA	AS TESTING DATA	
[30]	yes	no	no
[23]	yes	no	only subgroup accuracy
[9]	yes	yes	no
[24]	yes	no	no
[31]	yes	no	no
[32]	yes	no	no
Our work	yes	yes	yes

We are the first to extensively study the differences of applying data generated by these two families types of data synthesizing algorithms in end-to-end machine learning pipelines for utility and multiple fairness metrics.

## 3. Preliminaries

In this section we introduce the concepts of differential privacy and algorithmic fairness. We refer the reader to [1, 33, 34] for detailed explanation of these concepts. Additionally, we describe the synthetic data generation techniques and the datasets used in our experiments.

### 3.1 Differential privacy

Differential privacy is a rigorous privacy notion used to protect an individual’s data in a dataset disclosure. We present in this section notation and definitions that we will use to describe our privatization approach. We refer the reader to [1, 35, 36] for detailed explanations of these definitions and theorems.

PURE DIFFERENTIAL PRIVACY. A randomized mechanism M:D→A with data base domain D and output set A is *ϵ*-differentially private if, for any output A⊆Y and neighboring databases $D,D^{\prime }\in D$ (i.e., *D* and *D*^′^ differ in at most one entry), we have

$$Pr\left[M\left(D\right)\in A\right]\leq e^{\epsilon }\text{Pr}\left[M\left(D^{\prime }\right)\in A\right]$$

APPROXIMATE DIFFERENTIAL PRIVACY. A randomized mechanism M:D→A with data base domain D and output set A is (*ϵ*, *δ*)-differentially private if, for any output A⊆Y and neighboring databases $D,D^{\prime }\in D$ (i.e., *D* and *D*^′^ differ in at most one entry), we have

$$Pr\left[M\left(D\right)\in A\right]\leq e^{\epsilon }\text{Pr}\left[M\left(D^{\prime }\right)\in A\right]+\delta$$

The privacy loss of the mechanism is defined by the parameter *ϵ* ≥ 0 in the case of *pure* differential privacy and parameters *ϵ*, *δ* ≥ 0 in the case of *approximate* differential privacy.

The definition of neighboring databases used in this paper is user-level privacy. User-level privacy defines neighboring to be the addition or deletion of a single user in the data and all possible records of that user. Informally, the definition above states that the addition or removal of a single individual in the database does not provoke significant changes in the probability of any differentially private output. Therefore, differential privacy limits the amount of information that the output reveals about any individual.

A function *f* (also called query) from a dataset D∈D to a result set A⊆A can be made differentially private by injecting random noise to its output. The amount of noise depends on the sensitivity of the query [1].

### 3.2 Fairness metrics

In this section we present the definition of two different fairness metrics: Equal Opportunity [33] and Statistical Disparity [34]. Given a dataset *W* = (*X*, *Y*^′^, *C*) with binary protected attribute *C* (e.g. race, sex, religion, etc), remaining decision variables *X* and predicted outcome *Y*^′^, we define Equal Opportunity and Statistical Disparity as follows.

EQUAL OPPORTUNITY/ EQUALITY OF ODDS requires equal True Positive Rate (TPR) across subgroups:

$$Pr\left(Y^{\prime }=1|Y=1,C=0\right)=\text{Pr}\left(Y^{\prime }=1|Y=1,C=1\right)$$

where Y’ is the model output.

STATISTICAL PARITY requires positive predictions to be unaffected by the value of the protected attribute, regardless of true label

$$Pr\left(Y^{\prime }=1|C=0\right)=\text{Pr}\left(Y^{\prime }=1|C=1\right)$$

We follow the approach of [37, 38] and utilize difference in Equal Oportunity (DEO) = $|\text{Pr}\left(Y^{\prime }=1|Y=1,C=0\right)-\text{Pr}\left(Y^{\prime }=1|Y=1,C=1\right)|$ and difference in Statistical Parity (DSP) = $|\text{Pr}\left(Y^{\prime }=1|C=0\right)-\text{Pr}\left(Y^{\prime }=1|C=1\right)|$ to measure model fairness.

### 3.3 Differentially private synthetic data generators

We use several differentially private (DP) synthetic data generators that have been specifically tailored for generating tabular data with the goal of enhancing their utility for learning tasks. We consider two broad categories of approaches: i) marginal-based methods, ii) and Generative Adversarial Network (GAN) based models. In this section we provide an overview of each differentially private synthetic data generation algorithm used in our experiments. We refer the reader to [12–15, 17, 18, 39] for a detailed explanation of each one of these algorithms.

#### 3.3.1 Marginal-based methods

*MWEM PGM* [13]. Is a variation of the multiplicative weights with exponential mechanism algorithm (MWEM), which is an algorithm that generated synthetic data based on linear queries. The algorithm aims to produce a data distribution that produces query answers similar answers resulted when querying the real dataset. The MWEM PGM variation combines probabilistic graphical models (PGMs) with the MWEM algorithm. The structure of the graphical model is determined by the measurements, such that no information is lost relative to a full contingency table representation.

*MST* [12]. Is a synthetic data generation algorithm that acts selecting 2- and 3-way marginals for measurement. It combines one principled step, which is to find the maximum spanning tree (MST) on the graph where edge weights correspond to mutual information between two attributes, with some additional heuristics to ensure that certain important attribute pairs are selected, and a final step to select triples while keeping the graph tree-like.

*AIM* [14]. The Adaptive and interactive mechanism (AIM) for synthetic data generation is a variation of the MWEM PGM algorithm that innovates in the way it selects the most useful measurements. The ability to produce data with lower error, in comparison to MWEM PGM, is because of the new proposed features in the select stage, which defines a quality score that helps determine the private selection of the next best marginal to measure. The quality score takes into account factors such as the current measure of the candidate marginal, expected improvement, relevance to the workload, and available privacy budget. The algorithm also includes other techniques like adaptive selection of rounds and budget-per-round, as well as intelligent initialization.

*PrivBayes* [15]. In order to improve the utility of the generated synthetic data, [15] approximates the actual distribution of the data by constructing a Bayesian network using the correlations between the data attributes. This allows them to factorize the joint distribution of the data into marginal distributions. Next, to ensure differential privacy, noise is injected into each of the marginal distributions and the simulated data is sampled from the approximate joint distribution constructed from these noisy marginals.

#### 3.3.2 GAN-based methods

Generative neural networks (GANs) are a type of artificial neural network used in machine learning for generating new data samples similar to a given training dataset. Generative adversarial networks are based on a game, in the sense of game theory, between two machine learning models, a discriminator model *D* and the generator *G* model. The goal of the generator is to learn realistic samples that can fool the discriminator, while the goal of the discriminator is to be able to tell generator generated samples from real ones [16].

**Conditional Tabular GAN (CTGAN) [39]** is an approach for generating tabular data. CTGAN adapts GANs by addressing issues that are unique to tabular data that conventional GANs cannot handle, such as the modeling of multivariate discrete and mixed discrete and continuous distributions. It achieves these challenges by augmenting the training procedure with mode-specific normalization, and by employing a conditional generator and training-by-sampling that allows it to explore discrete values more evenly. When applying differentially private SGD (DP-SGD) [40] in combination with CTGAN the result is a DP approach for generating tabular data.

The **PATE (Private Aggregation of Teacher Ensembles)** framework [41] protects the privacy of sensitive data during training, by transferring knowledge from an ensemble of teacher models trained on partitions of the data to a student model. To achieve DP guarantees, only the student model is published while keeping the teachers private. The framework adds Laplacian noise to the aggregated answers from the teachers that are used to train the student models. CTGAN can provide differential privacy by applying the PATE framework. We call this combination PATE-CTGAN, which is similar to PATE-GAN [18], for images. The original dataset is partitioned into *k* subsets and a DP teacher discriminator is trained on each subset. Further, instead of using one generator to generate samples, *k* conditional generators are used for each subset of the data.

### 3.4 Datasets

We now describe the datasets used in our work. These datasets are commonly used in the literature for benchmarking algorithmic fairness in classification tasks [21, 42, 43].

#### 3.4.1 Adult dataset

In the Adult dataset (32561 instances), the features were categorized as protected variable (C): gender (male, female); and response variable (Y): income (binary); decision variables (X): the remaining variables in the dataset. We map into categorical variables all continuous variables.

#### 3.4.2 Prison recidivism dataset

From the COMPAS dataset (7214 instances), we select severity of charge, number of prior crimes, and age category to be the decision variables (X). The outcome variable (Y) is a binary indicator of whether the individual recidivated (re-offended), and race is set to be the protected variable (C). We utilize a reduced set of features as proposed in [21].

#### 3.4.3 Fair prison recidivism dataset

We construct a “fair” dataset based on the COMPAS recidivism dataset by employing a data preprocessing technique for learning non-discriminating classifiers from [44], which involves changing the class labels in order to remove discrimination from the dataset. This approach selects examples close to the decision boundary to be either ‘promoted’, i.e label flipped to the desirable class, or ‘demoted’, i.e label flipped to the undesirable class (ex: the ‘recidivate’ label in the COMPAS dataset is the undesirable class). By flipping an equal number of positive and negative class examples, the class skew in the dataset is maintained.

## 4. Results

**Fig 1:**
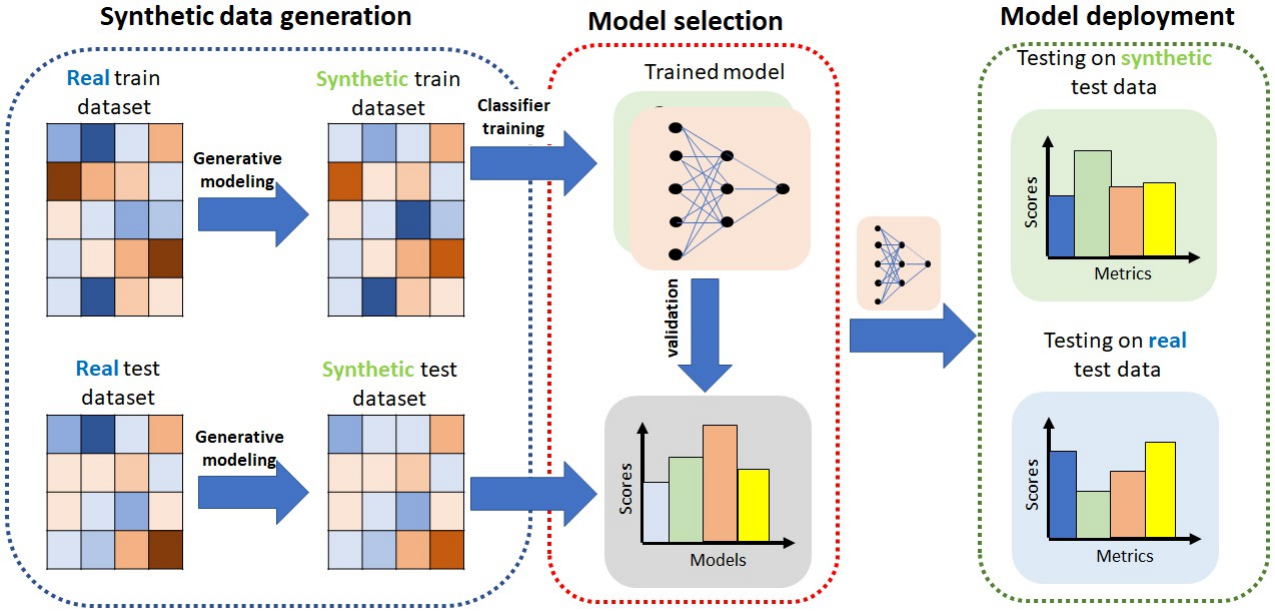
Pipeline for model training and evaluation using synthetic data (1) We generate Synthetic datasets for model training and model testing utilizing differentially private synthesizers. (2) We train models utilizing differentially private synthetic data and evaluate on a differentially private synthetic test data. Model selection is made during this phase. (3) Based on the previous phase results, model is trained using synthetic data and deployed. Model is applied to real (test) data in production phase.

One potential outcome of synthetic data sharing is the utilization of synthetic data for training and evaluating an ML model. The trained model could be deployed without assessing its performance on real data, due to lack of data access. However, it is important to acknowledge that these trained models are ultimately applied to real data. This scenario is illustrated in Fig 1. In our experiments, we address the concern that there may be substantial disparities in performance between the evaluation phase (employing synthetic data) and the deployment phase (utilizing real data). We refer to the experiments emulating the evaluation phase as *train on synthetic, test on synthetic (TSTS)*, and the experiments emulating the deployment phase as *train on synthetic, test on real (TSTR)*. We compare the performance of machine learning models trained with differentially private synthesizers, focusing on two performance dimensions: utility and fairness. We follow the approach of [23] and use logistic regression for downstream classification evaluation to avoid another layer of stochasticity. The utilization of a linear model allows us to better focus on the effects of different synthetic data generators in algorithmic fairness and model utility and reduce the effect of randomness in the training algorithms.

To assess the utility performance, we employ the AUC-ROC metric, which quantifies trade-off between the recall and false positive rate. We examine fairness performance through three different perspectives. Previous research [20] has indicated that differentially private machine learning models tend to perform worse on minority groups. To this point we evaluate the decay in accuracy for the different subgroups in the protected attribute. We also measure the difference in equality of odds (DEO) and the difference in statistical parity (DSP). These metrics allow us to assess any disparities or bias in the model’s predictions across different groups. Furthermore, we also investigate the extent to which one can accurately assess a model utilizing synthetic datasets. Again, we evaluate two performance dimensions: utility and fairness.

We utilized multiple differentially private marginal-based synthesizers (AIM, MST, MWEM-PGM, and PrivBayes) as well as GAN-based synthesizers (DP-GAN, DP-CTGAN, PATE-GAN, and PATE-CTGAN) to generate synthetic data. In our experiments, we generated datasets utilizing each synthetic data generation technique in combination with four different privacy-loss budgets *ϵ* = {0.5, 1.0, 5.0, 10.0}. The privacy-loss budget quantifies the privacy risk associated with the publication of the synthetic data set, as defined in section 3.1. The choice of these budgets is based on previous research in synthetic data analysis and published synthetic datasets [23, 27]. Previous studies showed that budgets at and lower than *ϵ* = 0.1 [17, 23] result in synthetic data with very low utility, so our experiments focused on budgets greater than 0.5. The selection of *ϵ* = 10.0 as the maximum budget aligns with other works in the literature on differentially private synthetic data generation [9, 11, 23]. We also observed this magnitude of privacy-loss budget in published synthetic datasets, such as the Global victim-perpetrator synthetic data, which was generated with a privacy-loss budget of *ϵ* = 12 [27].

We divide the real dataset into 10 random 80/20 data splits, separating the data into generator and test datasets. For the TSTR experiments, we run 10 rounds of synthetic DP data generation on the 80% splits (generator data), used to generate the synthetic train datasets. We use the remainder 20% split as test data in the TSTR experiments. For the TSTS experiments, we run 10 rounds of synthetic DP data generation on the 80% splits (generator data), where we generate synthetic train datasets. We use the same generator data to generate the synthetic test data used in the TSTs experiments. We utilize the SmartNoise Library [45] and DiffPrivLib [46] implementations of the synthesizers, and approximate-DP approaches use the library’s default value of *δ*.

We train Logistic Regression models using the generated DP synthetic train datasets. In experiments where we test the trained models on real data, model performance is evaluated on the real test data (the 20% test split from the real data). In experiments where we test the trained models on synthetic data, models are evaluated using the synthetic test datasets.

We report, for each technique and each value of privacy loss parameter, the mean across 10 rounds. The mean across multiple rounds serve to capture the behavior of each synthesizer and attenuate the effects of randomness. A similar approach was used in [23]. Our experiments use three datasets: the UCI Adult dataset [47] and ProPublica’s COMPAS recidivism data [48], and a fair COMPAS dataset as defined in Section 3.4. The fair COMPAS dataset provides a way to evaluate synthetic data generation performance in fair and biased versions of the same dataset.

### 4.1 Utility analysis: Impacts of synthetic data in machine learning pipelines

We evaluate the quality of models trained with synthetic datasets by measuring AUC and accuracy of the protected class. We consider privacy-loss budgets of *ϵ* = 0.5, 1.0, 5.0 and 10.0. We compare the AUC obtained in our experiments with the AUC measured by training models with the real (non-synthetic) Adult, COMPAS, and fair COMPAS datasets.

**Fig 2:**
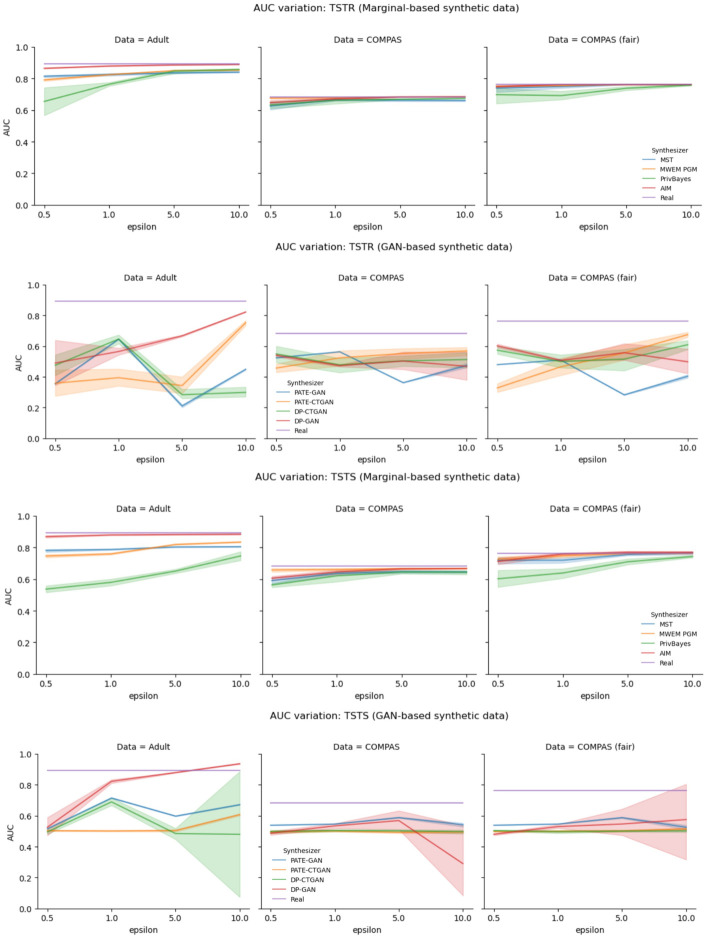
Impact in utility caused by the use of differentially private synthetic data in model training and testing. In the first two rows we show the decay in model utility when utilizing marginal-based and GAN-based synthetic datasets for model training. In the third and fourth rows we show what is the measured model utility when the instrument for measuring model performance is a synthetic dataset.

Fig 2 shows AUC for different privacy losses and different synthesizers. The plots show the variation of AUC as a function of privacy-loss parameter *ϵ* for marginal-based and GAN-based synthesizers. The first row refers to marginal-based synthesizers in the TSTR mode. Experiments with COMPAS and fair COMPASS datasets showed that models trained on marginal-based synthetic data perform similarly to the baseline model (trained on real data). For all four synthesizers, we see an increase in AUC as we increase *ϵ*. Experiments with Adult dataset showed that AIM synthesizer outperformed all other synthesizer in both experimental settings: TSTR and TSTS. For COMPAS dataset (which has a small dimension) the performance of marginal-based synthetic datasets as training data is very close to the performance of the real data. The second row of Fig 2 presents the performance of GAN-based synthetic data. Overall, the performance of GAN-based synthesizer is worse and the performance of the marginal-based synthesizer. The utility of data produced by GAN-based synthesizers fluctuated as we increased privacy-loss budget. This phenomenon had been previously observed in [17]. With AUC mostly fluctuating around ≈ 0.5, we can say that GAN-based synthetic data do not do much better than random guessing (for various values of *ϵ*). We attribute the fluctuations to the fact that GAN-based synthesizers are known to be data hungry and not capture well the intrinsic relationships between features when using small data sets for training data synthesizers [49]. The inferior performance of GAN-based synthesizers was also noted by [30], which showed that models trained on GAN-based synthetic data perform worse than models trained on marginal-based synthetic data.

In third and fourth rows of Fig 2 we present the plots of variation of AUC for different values of epsilon for TSTS models. The plots in the third row refer to performance of models trained on marginal-based synthesizers, the the plots in the fourth row refer to GAN-based synthesizers. By comparing the models trained with marginal-based synthetic data when evaluated in different modes—TSTR and TSTS, we see that the assessment is very similar in both cases when the synthesizers are MST, AIM and MWEM PGM. When assessing with synthetic data, we notice that PrivBayes present a large difference in assessment results when assessing model trained on Adult and fair COMPAS synthetic data. GAN-based synthetic data, once again, present inconsistent behavior when used for model assessment. When comparing the assessments TSTR and TSTS, we notice that using DP-GAN sythetic data for model assessment can over estimate model AUC. Overall, GAN-based synthetic data made assessments that are as good as random guessing.

### 4.2 Fairness analysis: Impacts of synthetic data in machine learning pipelines

#### 4.2.1 Impacts on subgroup accuracy

In the previous section, we showed that adding privacy by utilizing synthetic datasets in machine learning pipelines results in a utility decrease in most cases. We now proceed to perform a fairness analysis. In the first experiment, presented in Table 2, we analyzed model accuracy for different groups in the protected class. The goal of the experiment is to understand whether the addition of privacy to the data pipeline harms model utility more for the minority class than it does for the privileged class. Results in Table 2 refer to the Adult, COMPAS and COMPAS (fair) datasets.

**Table 2:** Accuracy comparison for different subgroups of the protected attribute. The comparison presented accounts for synthetic data generated with privacy-loss parameter *ϵ* = 5.0. We show a comparison of model accuracy for the different groups measured with real data (R), and model accuracy measured with synthetic data (S).

	ACCURACY OF DIFFERENT SUBGROUPS			
SYNTHESIZER	minority (R)	minority (S)	privileged (R)	privileged (S)
	ADULT DATA			
Real	0.924	–	0.804	–
AIM	0.919	0.916	0.794	0.807
MWEM PGM	0.909	0.898	0.779	0.770
MST	0.914	0.895	0.756	0.765
PrivBayes	0.892	0.713	0.709	0.648
DP-GAN	0.733	0.929	0.585	0.855
PATE-CTGAN	0.892	0.938	0.695	0.942
DP-CTGAN	0.889	0.999	0.693	0.999
PATE-GAN	0.892	0.874	0.695	0.854
	COMPAS DATA			
Real	0.632	–	0.644	–
AIM	0.630	0.610	0.645	0.633
MWEM PGM	0.630	0.627	0.644	0.598
MST	0.616	0.614	0.631	0.616
PrivBayes	0.619	0.598	0.639	0.622
DP-GAN	0.497	0.514	0.451	0.452
PATE-CTGAN	0.536	0.497	0.377	0.499
DP-CTGAN	0.499	0.463	0.527	0.450
PATE-GAN	0.466	0.370	0.624	0.422
	COMPAS (FAIR) DATA			
Real	0.690	–	0.679	–
AIM	0.690	0.693	0.679	0.701
MWEM PGM	0.690	0.678	0.679	0.707
MST	0.691	0.685	0.704	0.699
PrivBayes	0.674	0.632	0.672	0.656
DP-GAN	0.513	0.366	0.542	0.474
PATE-CTGAN	0.471	0.499	0.437	0.510
DP-CTGAN	0.491	0.524	0.489	0.528
PATE-GAN	0.528	0.389	0.562	0.442

We first note that the model accuracy decay when training models with marginal-based (AIM) Adult synthetic data is smaller for the minority subgroup (Female), which presented an accuracy decay of 0.005, than it is for the privileged subgroup (Male), which presented an accuracy decay of 0.01. Models trained with marginal-based COMPAS synthetic data presented a slightly larger accuracy decay for the minority subgroup (Black) when compared to the accuracy decay for the privileged subgroup (Caucasian). Models trained on synthetic COMPAS fair dataset did not show accuracy decay in any of the subgroups. Overall, marginal-based synthesizers do not further accentuate subgroup accuracy disparities.

In the case of models trained with GAN-based synthetic datasets, no clear pattern of subgroup accuracy disparity was observed. For models trained with GAN-based Adult synthetic data, accuracy decay of the minority class (Female) was smaller than accuracy decay for the privileged class (Male). In the case of models trained with GAN-based COMPAS and COMPAS (fair) synthetic data, accuracy of both subgroups were close to 0.5, confirming previous results, that showed model trained with GAN-based data acting like random classifiers. What we confirmed with this experiment is that this phenomenon happens for all subgroups.

#### 4.2.2 Impacts on statistical parity

A model presents statistical parity if the percentage of positive predictions are the same for all subgroups. The goal of the experiments in this section is to measure whether models trained with synthetic data preserve the characteristics of models trained on real data.

Our experiments measure the difference in statistical parity (DSP) of models. We measure DSP of models using real data—DSP(R), and using synthetic data—DSP(S). We present a detailed comparison of DSP for all three datasets and all synthesizers on Table 3. We notice from our experiments that several models trained on synthetic data seem to be less biased than the model trained on real data. In terms of training models that performs similarly to models trained with real data, AIM synthesizer outperformed all other algorithms, followed by MWEM PGM synthesizer. AIM presented the best results in preserving statistical parity, based on experiments with all three datasets: Adult, COMPAS and COMPAS fair. GAN-based synthesizers, overall presented an intriguing performance: in some cases it seems like it has achieved perfect fairness.

**Table 3:** Difference in statistical parity (DSP) of models trained with synthetic data. We measure the DSP of models using real test data—DSP(R) and synthetic test data DSP(S). DEO delta quantifies the difference between DSP(R) and DSP(S). All synthetic data where generated using privacy-loss parameter *ϵ* = 5.0.

DATA	SYNTHESIZER	DSP(R)	DSP(S)	DSP delta
Adult	AIM	0.193	0.184	0.009
	MST	0.083	0.072	0.011
	MWEM PGM	0.168	0.159	0.009
	PrivBayes	0.051	0.043	0.008
	DP-CTGAN	-0.001	0.000	-0.001
	DP-GAN	0.346	0.253	-0.093
	PATE-CTGAN	0.000	0.000	0.000
	PATE-GAN	0.000	0.000	0.000
	Real	**0.189**		
COMPAS	AIM	-0.207	-0.204	-0.002
	MST	-0.182	-0.101	-0.082
	MWEM PGM	-0.218	-0.190	-0.028
	PrivBaeys	-0.211	-0.153	-0.058
	DP-CTGAN	-0.034	0.001	-0.034
	DP-GAN	0.072	-0.089	0.161
	PATE-CTGAN	-0.008	-0.009	0.001
	PATE-GAN	0.000	-0.001	0.001
	Real	**-0.205**		
COMPAS (fair)	AIM	0.009	0.020	-0.010
	MST	-0.185	-0.090	-0.095
	MWEM PGM	-0.018	0.015	-0.032
	PrivBayes	-0.065	0.005	-0.060
	DP-CTGAN	-0.034	-0.004	-0.030
	DP-GAN	0.066	0.096	-0.030
	PATE-CTGAN	0.000	0.000	0.000
	PATE-GAN	0.000	0.000	0.000
	Real	**-0.025**		

To understand better what is behind this apparent fairness provided some GAN-based synthetic datasets, we investigate the percentage of positive labelled samples in the training data, evaluation data and predictions of models on TSTR and TSTS modes. We present percentages for minority and privileged classes in Table 4.

**Table 4:** Ratio of samples with positive labels for each subgroup in the protect class in the Adult, COMPAS and COMPAS (fair) datasets. We compare percentages present in the true labels of the real data and the predicted labels. Analogously, we measure the ratio of samples with positive label present in the synthetic generated data and predicted labels for datasets generated using distinct synthesizer techniques. Predictions(R) represents ratio of positive prediction labels of an experiment where model trained on synthetic data was evaluated on real data, and Predictions(S) ratio of positive prediction labels of an experiment where model trained on synthetic data was evaluated on synthetic data.

	RATIO OF POSITIVE LABELS					
SYNTHESIZER	GENERATED DATA		PREDICTIONS(R)		PREDICTIONS(S)	
	ADULT DATA					
	Female	Male	Female	Male	Female	Male
Real	0.109	0.303	0.055	0.244		
AIM	0.110	0.303	0.049	0.242	0.056	0.239
MWEM PGM	0.120	0.307	0.042	0.209	0.043	0.202
MST	0.123	0.297	0.032	0.115	0.031	0.102
PrivBayes	0.259	0.342	0.004	0.060	0.102	0.143
PATE-GAN	0.125	0.144	≈ 0	≈ 0	≈ 0	≈ 0
PATE-CTGAN	0.056	0.058	≈ 0	≈ 0	≈ 0	≈ 0
DP-GAN	0.061	0.307	0.199	0.545	0.016	0.269
DP-CTGAN	≈ 0	0.002	0.227	0.130	≈ 0	≈ 0
	COMPAS DATA					
	Black	Caucasian	Black	Caucasian	Black	Caucasian
Real	0.504	0.402	0.499	0.294		
AIM	0.503	0.405	0.504	0.297	0.500	0.297
MWEM PGM	0.504	0.403	0.514	0.294	0.498	0.302
MST	0.477	0.443	0.567	0.384	0.538	0.433
PrivBayes	0.489	0.436	0.566	0.352	0.550	0.387
PATE-GAN	0.231	0.196	0.397	≈ 0	≈ 0	≈ 0
PATE-CTGAN	0.548	0.541	0.715	0.975	0.981	0.949
DP-GAN	0.745	0.583	0.442	0.908	0.004	≈ 0
DP-CTGAN	0.471	0.455	0.302	0.218	0.217	0.179
	COMPAS (FAIR) DATA					
	Black	Caucasian	Black	Caucasian	Black	Caucasian
Real	0.454	0.493	0.488	0.463		
AIM	0.453	0.493	0.487	0.487	0.478	0.492
MWEM PGM	0.454	0.491	0.480	0.463	0.466	0.478
MST	0.485	0.446	0.495	0.310	0.478	0.393
PrivBayes	0.450	0.497	0.561	0.491	0.530	0.520
PATE-GAN	0.232	0.194	0.397	≈ 0	≈ 0	≈ 0
PATE-CTGAN	0.606	0.598	0.397	≈ 0	≈ 0	≈ 0
DP-GAN	0.593	0.664	0.560	0.836	0.865	0.744
DP-CTGAN	0.581	0.576	0.492	0.398	0.421	0.401

As we investigate GAN-based synthetic data, we observe in Table 4 that synthetic data generated with PATE-GAN and PATE-CTGAN presents very similar percentages of samples with positive labels for each subgroup that belongs to the protected attribute. At a first sight, this seems like a dataset with promising fairness capabilities. However, when training models with such data, in most cases there were no positive predictions resulting from the model scoring. The model trained with PATE-GAN and PATE-CTGAN data acts like a majority baseline classifier for all groups. The datasets generated with DP-CTGAN presented an accentuated disparity in positive labels percentages between minority and privileged classes. In the real Adult data 30% of privileged class contains positive labels, while only 10% of minority class contains positive labels. Although DP-GAN synthesizer generates data where 31% of privileged class with positive labels (a value similar to the one presented in the real data—30%), there is a significant decrease in the percentage of positive class in the minority class, which is ≈6%. This imbalance is even further accentuated by the models trained with DP-GAN synthetic data. Model predictions resulted in over half of samples from the privileged class being classified with positive labels (versus 20% of minority class). For models trained with COMPAS and COMPAS fair synthetic datasets, similar behavior was observed.

AIM once again was the best overall performing model, as it preserves similar percentages of positive labels for all groups, 11% and 30% (compared to 11% and 30% in real data). Models trained with AIM also presented similar metric to models trained with real data, and even presenting slightly improvement in fairness. The runner-up synthetic data generator in preserving the ratio of positive labels was the MWEM algorithm.

The DSP delta presented in Table 3 quantifies the difference in DSP observed during model evalution with real data and model evaluation with synthetic data. For Adult dataset, a positive DSP delta means that evaluation with synthetic data observed fairer results than evaluation with real data. For COMPAS and fair COMPAS data, a negative DSP delta means that evaluation with synthetic data observed fairer results than evaluation with real data.

Across all datasets, models trained with AIM and MWEM PGM presented DSP metrics very similar to models trained with real data, this is captured by the DSP(R) metric.

#### 4.2.3 Impacts on equal opportunity

Equal Opportunity requires equal True Positive Rate (TPR) across subgroups. Difference in equal opportunity (DEO) measures the difference of privileged group TPR and minority group TPR.

We perform a thorough analysis to understand two points related to equal opportunity. First, what is the DEO of models trained with synthetic datasets, and how does it compare with models trained with real data? Second, given that true positive rate is the foundation for understanding equal opportunity, we investigate whether synthetic data preserves true positive rates across all subgroups.

We present in Table 5 experiment results comparing DEO of models trained with differentially private synthetic datasets (*ϵ* = 5.0). These experiment are similar to the statistical parity experiments, we use real data—DEO(R)—to measure DEO of models trained on synthetic data, as well as synthetic data—DEO(S).

**Table 5:** Difference in equal opportunity (DEO) of models trained with synthetic data. We measure the DEO of models using real test data—DEO(R) and synthetic test data DEO(S). DEO delta quantifies the difference between DEO(R) and DEO(S). All synthetic data where generated using privacy-loss parameter *ϵ* = 5.0.

DATA	SYNTHESIZER	DEO (R)	DEO (S)	DEO delta
Adult	AIM	0.209	0.200	0.009
	MST	0.038	0.076	-0.037
	MWEM PGM	0.206	0.200	0.006
	PrivBayes	0.094	0.026	0.067
	DP-CTGAN	-0.002	≈0.00	-0.002
	DP-GAN	0.527	0.641	-0.116
	PATE-CTGAN	0.000	0.000	0.000
	PATE-GAN	0.000	0.000	0.000
	Real	**0.173**		
COMPAS	AIM	-0.201	-0.195	-0.006
	MST	-0.150	-0.089	-0.061
	MWEM PGM	-0.215	-0.224	0.009
	PrivBayes	-0.177	-0.127	-0.051
	DP-CTGAN	-0.031	-0.000	-0.031
	DP-GAN	-0.075	0.020	0.055
	PATE-CTGAN	-0.011	-0.009	-0.002
	PATE-GAN	0.000	-0.001	0.001
	Real	**-0.204**		
COMPAS (fair)	AIM	0.007	0.013	-0.006
	MST	-0.181	-0.073	-0.107
	MWEM PGM	-0.019	0.037	-0.056
	PrivBayes	-0.057	0.005	-0.062
	DP-CTGAN	-0.030	-0.005	-0.026
	DP-GAN	0.097	0.087	0.010
	PATE-CTGAN	0.000	0.000	0.000
	PATE-GAN	0.000	-0.001	-0.000
	Real	**-0.027**		

Model trained with AIM and MWEM PGM synthetic data were the only ones that presented a similar DEO to the baseline model, outperforming all other models trained with synthetic data. Note that our comparison, as in the DSP case, focus on understanding which synthetic datasets can train models that behave as close as possible to models trained with real data. Models trained with MST, which presented promising utility metrics and subgroup accuracy, did not capture as well the difference in equality on odds in experiments with the Adult data. For experiments with COMPAS and fair COMPAS data, MST performs better, but still worse than AIM and MWEM PGM, as we can see on Table 5.

**Fig 3:**
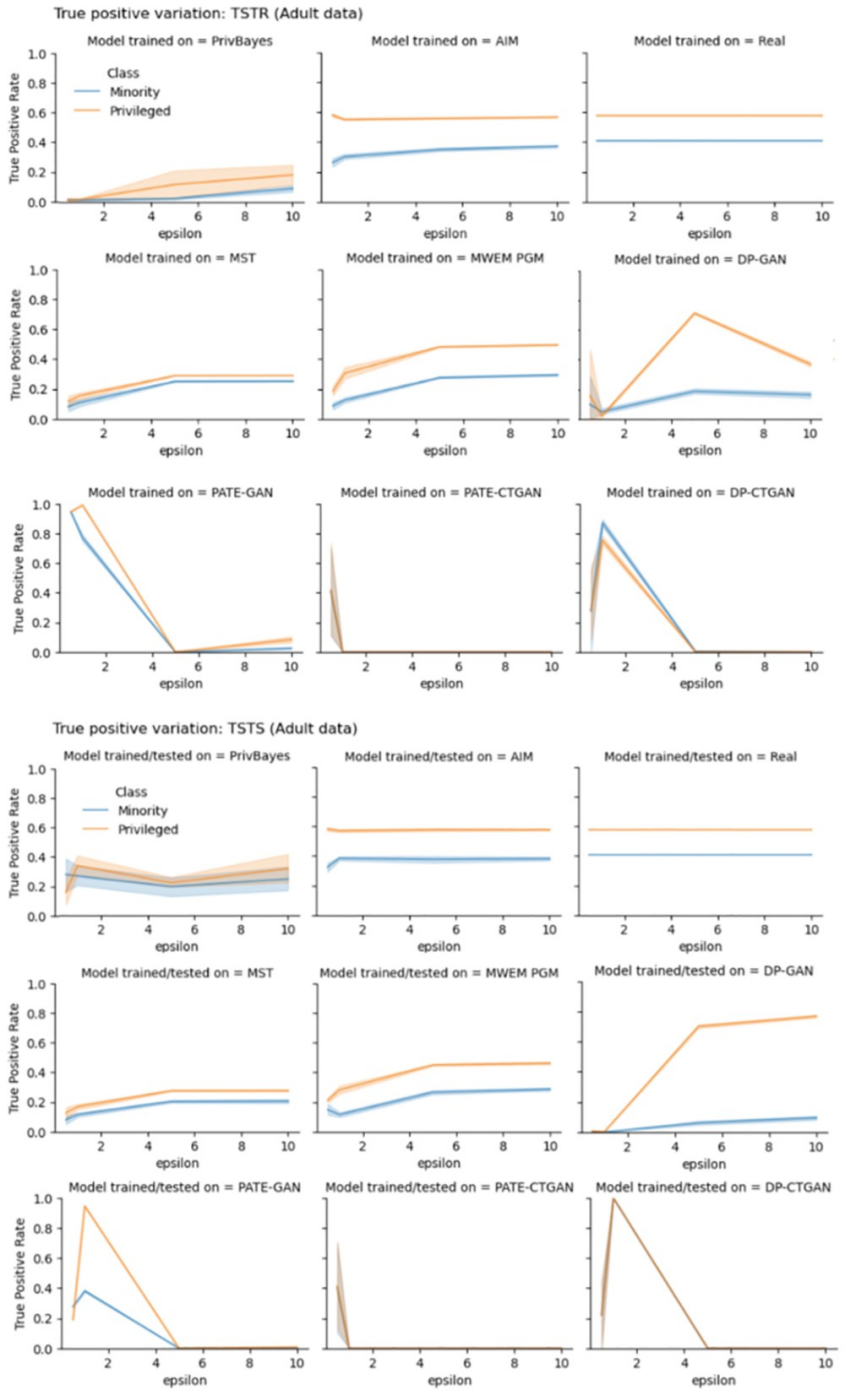
True positive rate (TPR) variation of different subgroups of the protected attribute of the Adult data. The top three rows shows TPR variation for different values of privacy-loss parameter *ϵ*, TSTR mode. The bottom three rows shows TPR variation for different values of privacy-loss parameter *ϵ*, TSTS mode.

**Fig 4:**
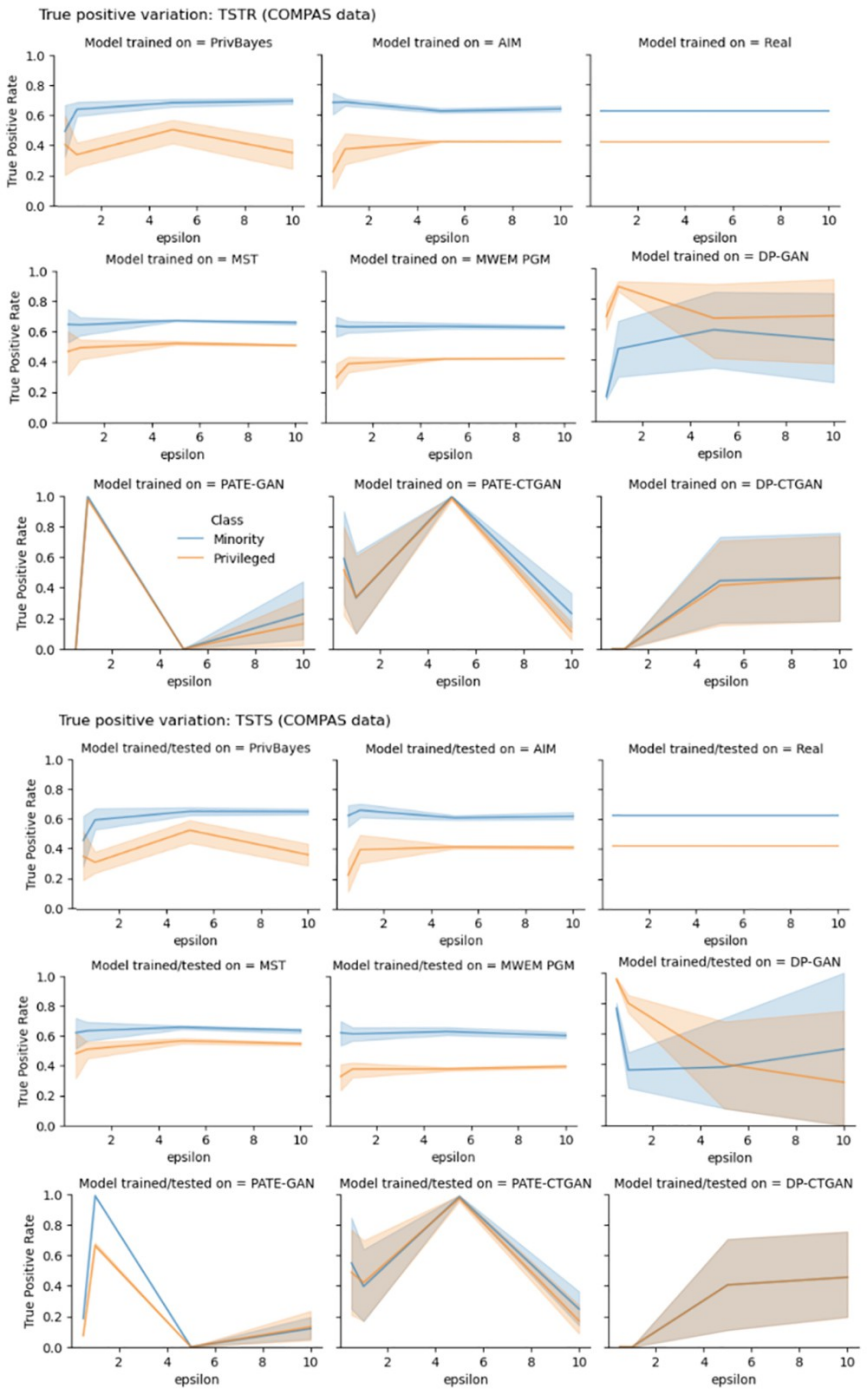
True positive rate (TPR) variation of different subgroups of the protected attribute of the COMPAS data. The top three rows shows TPR variation for different values of privacy-loss parameter *ϵ*, TSTR mode. The bottom three rows shows TPR variation for different values of privacy-loss parameter *ϵ*, TSTS mode.

**Fig 5:**
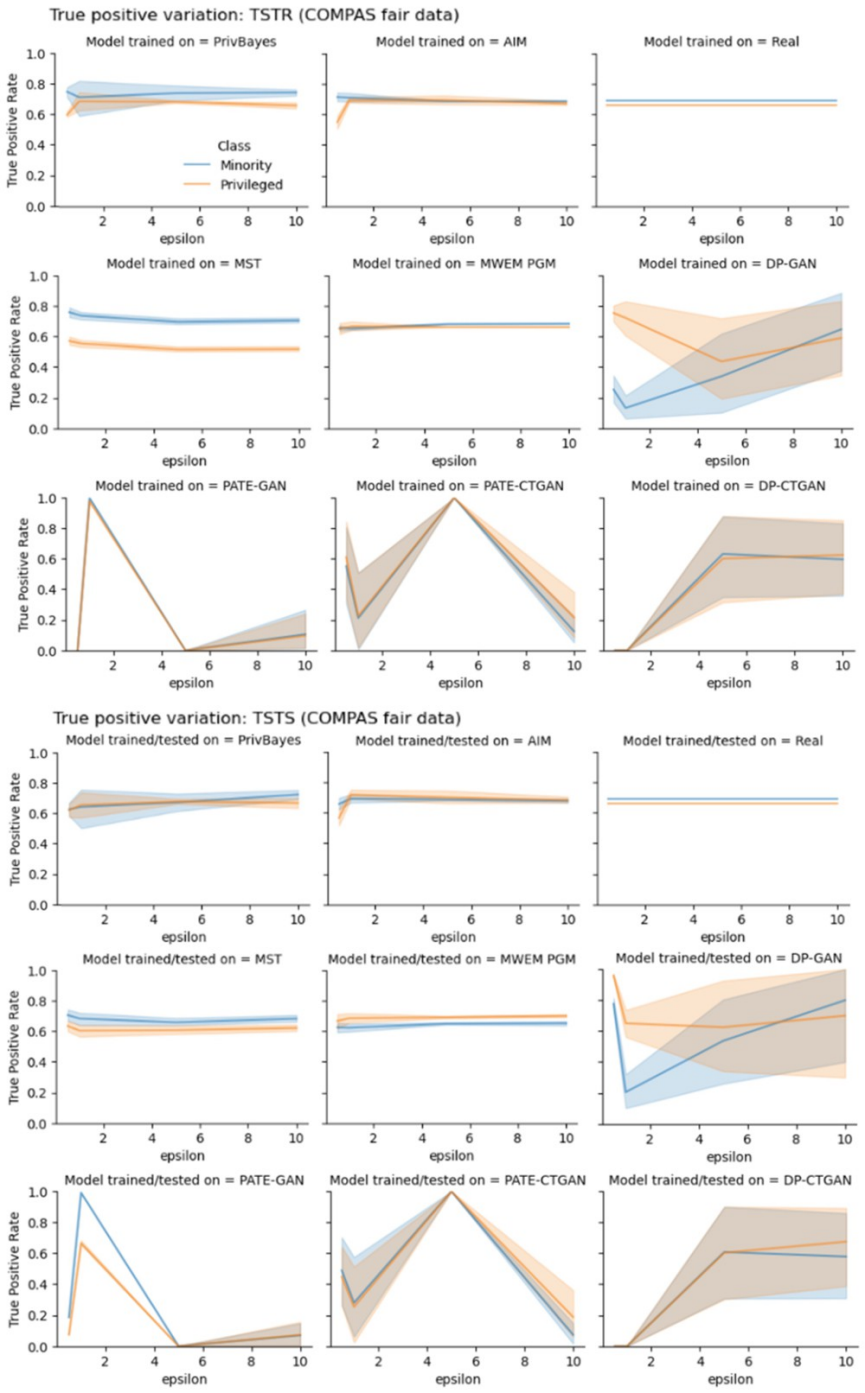
True positive rate (TPR) variation of different subgroups of the protected attribute of the COMPAS (fair) data. The top three rows shows TPR variation for different values of privacy-loss parameter *ϵ*, TSTR mode. The bottom three rows shows TPR variation for different values of privacy-loss parameter *ϵ*, TSTS mode.

As we investigate the details of variation in TPR it becomes clear AIM algorithm is the the best technique for training models that preserve fairness characteristics of models trained with real data, followed by MWEM PGM algorithm. Experiments with Adult data (Fig 3) show that the difference between the privileged group TPR and the minority group TPR of models trained with AIM data is very similar to the difference between subgroups TPR of models trained with real data, for all values of privacy-loss parameter *ϵ*. Similar conclusion is achieved by observing experiments with COMPAS and COMPAS fair data (Figs 4 and 5). Not only the difference between the subgroup TPR of the model trained with AIM and MWEM PGM synthetic data is close to that of the model trained with real data, but the true positive rates of the subgroups are also very similar to the TPR of the model trained with real data. Figs 3–5 show that models trained with marginal-based synthetic data outperforms models trained with GAN-based synthetic data for our tested datasets.

We make a similar analysis when evaluating how good synthetic datasets are for assessing TPRs. Figs 3–5 also present plots of TPR when synthetic data is used during model assessment. Models trained with AIM and MWEM PGM data present very similar assessment when using both real and synthetic data as test data. Models trained on MST and PrivBayes present greater discrepancies. Models trained on GAN-based data present even greater discrepancies between assessments made with real and synthetic data as test data.

## 5. Discussion

### 5.1 Marginal-based synthetic data does better at training and assessing utility of models

The results in section 4.1, showed that models trained marginal-based synthetic data can have similar performance to models trained on real data. We observed the AIM synthetic data generation algorithm generated data that performed very closely to real data when training and evaluating machine learning models. The AIM data synthesizer presented a consistent performance across all datasets and for all values of privacy-loss parameter *ϵ*. To showcase a clear comparison between marginal-based and GAN-based synthesizers, we ranked the utility performance of all synthesizers taking based on two criteria: ability to generate synthetic data for model training and ability to generate synthetic data for model assessment. We ranked the synthesizers for each dataset used in our experiments. Table 6 shows the ranking of synthesizers when generating training and assessment data for the Adult data, COMPAS data and COMPAS (fair) data. The table also shows a comparison of model AUC measured in TSTR mode—AUC(R), and model AUC measured in TSTS mode—AUC(S). All table results accounts for synthetic data generated with privacy-loss parameter *ϵ* = 5.0.

**Table 6:** Synthesizer utility comparison. We compare and rank all synthesizers by their ability to generate quality training data and evaluation data for machine learning pipelines. The comparison presented accounts for synthetic data generated with privacy-loss parameter *ϵ* = 5.0. In addition to present a performance ranking for Adult, COMPAS data and COMPAS (fair) data, we show a comparison of model AUC measured in TSTR mode—AUC(R), and model AUC measured in TSTS mode—AUC(S).

	ADULT		COMPAS		COMPAS FAIR	
SYNTHESIZER	RANK	AUC (R/S)	RANK	AUC (R/S)	RANK	AUC (R/S)
AIM	1st	0.886/0.882	2nd	0.683/ 0.666	2nd	0.761/0.771
MWEM PGM	2st	0.850/0.820	1st	0.684/ 0.666	1st	0.762/0.762
MST	3rd	0.836/0.804	4th	0.662/0.647	3rd	0.763/0.756
PrivBayes	4th	0.846/0.650	3rd	0.668/0.645	4th	0.738/0.710
DP-GAN	5th	0.667/0.880	7th	0.503/0.568	5th	0.557/0.546
PATE-CTGAN	6th	0.343/0.504	5th	0.552/0.492	6th	0.556/0.502
DP-CTGAN	7th	0.284/0.485	6th	0.504/0.502	7th	0.515/0.501
PATE-GAN	8th	0.210/0.597	8th	0.362/0.587	8th	0.283/0.588

Synthetic data generated with the AIM algorithm outperforms (or tie with) all other synthetic data for both tasks: utility as training data for machine learning models and utility as evaluation data for machine learning models. The performance of synthetic datasets generated with AIM was very similar to real data, both when using the synthetic data for model training and model assessment. For model training, when comparing the AUC achieved by model trained with the real Adult dataset (AUC = 0.892) to the metrics achieved by models trained with AIM Adult synthetic data (AUC = 0.886) and MWEM PGM Adult synthetic data (AUC = 0.850), the decrease in performance is small. The synthetic datasets also present a good performance as assessment data. The model assessment with AIM generated data showed good results, with an assessment of AUC = 0.882. Assessment with other marginal-based synthesizers, MST data (AUC = 0.804) and MWEM PGM data (AUC = 0.820), also presented consistent results, with a small decay. Although PrivBayes data presents good performance in model training (AUC = 0.846), there is a significant discrepancy between assessment utilizing real data and assessment utilizing synthetic data. We reached similar conclusions when analysing results for COMPAS and COMPAS (fair) data. Overall, our experiments using GAN-based data as training data resulted in models with utility very close to random guess. DP-GAN synthetic data performed slightly better than the rest of GAN-based datasets. We believe that the fact that the datasets used in our experiments are relatively small (less than 50k rows), GAN-based synthesizers do not have enough data samples to capture correctly the relationships between features. Although experiments with larger datasets can be useful to uderstand whether GAN-based synthesizers could do better with more data, the datasets used in our experiments are great representations of datasets found in the real world. Such datasets are rarely larger than a couple of thousand rows.

#### 5.2 Marginal-based synthetic data preserves and better assess model fairness

We evaluated the performance of the synthetic datasets based on two key model fairness tasks: the ability to mirror the behavior of actual data in downstream model fairness, and the ability to produce synthetic data for assessing model fairness. Our analysis includes a rigorous assessment of model fairness, which includes measuring subgroup accuracy, the difference in statistical parity(DSP) and the difference in equal opportunity (DEO). Beyond measuring the classical fairness metrics, we also assess the Positive Predictive Value (PPV) and True Positive Rate (TPR) for each subgroup within the protected class. The significance of evaluating PPV and TPR lies in understanding if the model upholds fairness because it accurately represents PPV and TPR for all subgroups, or if it does so merely by acting as a random classifier.

Table 7 shows the best synthesizers in end-to-end machine learning pipelines when evaluating for fairness metrics. All table results accounts for synthetic data generated with privacy-loss parameter *ϵ* = 5.0.

**Table 7:** Best synthesizers for each fairness metric evaluated in the experiments: Subgroup accuracy, difference in statistical parity and difference in equality of odds. We also present the synthesizers that best preserve PPV and TPR accross subgroups. We present the two best synthetic data generator for each task. We selected best synthesizer and runner up based on experiments with privacy-loss budget *ϵ* = 5.0.

METRIC	BEST SYNTHESIZER	RUNNER UP
Subgroup accuracy	AIM	MWEM PGM
Difference in statistical parity	AIM	MWEM PGM
Difference in equality of odds	AIM	MWEM PGM
PPV accross subgroups	AIM	MWEM PGM
TPR accross subgroups	AIM	MWEM PGM

Throughout fairness experiments we observed that marginal-based synthetic datasets performed better than GAN-based synthetic dataset across all algorithmic fairness metrics. AIM and MWEM PGM synthetic data generation algorithms not only outperformed all other synthetic data generation algorithms, but these synthesizers generated data that performed similarly to real data in the three fairness metrics, and in our deeper investigations on PPV an TPR. This advantage was observed for multiple values of privacy-loss parameter *ϵ*, when synthetic data was used as a training dataset as well as when used as a testing dataset.

The investigation of subgroup PPV and TPR metrics clarified our observations regarding model fairness performances. We note that AIM and MWEM PGM synthetic data presents a ratio of positive labels comparable to that obtained with real data (Table 4), for all subgroups. When evaluating the ratio of positive labels in prediction for all subgroups in the Adult data (female and male) and in the COMPAS and COMPAS (fair) data (black and caucasian) in Table 4, we see that AIM and MWEM PGM also results is metrics that are the closest to real data.

The evaluation of true positive rate provides more insights into the bias introduced by synthetic dataset in end-to-end machine learning pipelines. Figs 3–5 shows the variation of TPR for different values of *ϵ*, in experiments with Adult, COMPAS and fair COMPAS, respectively. For COMPAS dataset, AIM provides the best performance, comparable the real dataset in an end-to-end analysis. For Adult data, *ϵ* > 1 provides comparable metrics. Other algorithms, such as PrivBayes, that presented utility results (AUC metric) comparable to real data, showed low performance in terms of TPR. Finally, marginal-based synthesizers presented similar performance from the point of view of utility and fairness for both biased and fair versions of the COMPAS dataset.

### 5.3 Limitations and future work

Although the datasets utilized in our analysis are commonly employed in fairness literature, extending the validity of our findings to larger-scale datasets would provide a more comprehensive understanding of the generalizability and robustness of marginal-based synthetic data approaches. Future research should focus on exploring the performance of these frameworks in real-world scenarios with diverse and extensive datasets, such experiments would clarify whether synthesizers behave differently in the presence of different types of dataset. This would contribute to the broader applicability and reliability of synthetic data methods in various domains and facilitate a more nuanced understanding of their limitations and capabilities. Finally, our work focuses solely on classification tasks. Extending our analysis to regression tasks, and evaluating fairness metrics [50] in regression tasks when in presence of differentially private synthetic data hasn’t been studied yet and would be an interesting sequel to this work.

## 6. Conclusion

Our research comprehensively evaluates the impact of differentially synthetic datasets for training and testing machine learning pipelines in the case of tabular datasets. Specifically, we compare the performance of marginal-based and GAN-based synthesizers within a machine-learning pipeline and analyze various utility and fairness metrics for tabular datasets, across multiple values privacy-loss parameter *ϵ*.

Our main findings are as follows: Marginal-based synthetic data demonstrated comparable utility to real data in end-to-end machine-learning pipelines. AIM and MWEM PGM synthetic data generators provided the best utility across experiments, for various values of *ϵ*. AIM synthetic data, in particular, performed provided utility very close to models trained on real data, for multiple values of epsilon, for all datasets: Adult (AUC(R) = 0.892 vs AUC(S) = 0.886), COMPAS (AUC(R) = 0.684 vs AUC(S) = 0.683) and COMPAS fair (AUC(R) = 0.762 vs AUC(S) = 0.761). Furthermore, we show that model evaluation using synthetic data also provides similar results to evaluation using real data, for tabular data. The metrics obtained when utilizing AIM marginal-based synthetic data are comparable to real data, across all datasets and for multiple values of epsilon. Synthetic datasets trained with AIM and MWEM PGM synthetic data do not increase model bias and can provide a realistic fairness evaluation. Our study reveals that AIM and MWEM PGM synthetic data can train models that achieve similar utility and fairness characteristics as models trained with real data. Additionally, when used to evaluate the utility and fairness of machine learning models, our experiments showed that the synthetic datasets generated by the AIM algorithm exhibits behavior very similar to real data, for various values of *ϵ*.

One important point to raise is that, across all datasets used in our experiments (Adult, COMPAS and COMPAS fair) marginal-based algorithms (AIM and MWEM PGM specifically) were the best performing algorithms in terms of utility and fairness. From our experiments we gained evidence about an important fact: that synthesizer performance is independent from fairness characteristics of the original dataset.

These findings highlight synthetic data’s potential reliability and viability as a substitute for real datasets in end-to-end machine learning pipelines for tabular data. Furthermore, our research sheds light on the implications of model fairness when utilizing differentially private synthetic data for model training.

One crucial observation is that synthetic data that does well in model training might perform differently when used as evaluation data. This was the case with Privbayes and most of the GAN-based synthetic data generators. This observation is important as synthetic data techniques gain acceptance as a data publishing approach in domains such as healthcare, humanitarian action, education, and population studies.
